# Draft genome sequence of *Acinetobacter* sp. AYS6, a potential plant growth-promoting endophyte

**DOI:** 10.1128/MRA.00464-23

**Published:** 2023-09-22

**Authors:** Ayomide Emmanuel Fadiji, Ayansina Segun Ayangbenro, Akinlolu Olalekan Akanmu, Olubukola Oluranti Babalola

**Affiliations:** 1 Food Security and Safety Focus Area, Faculty of Natural and Agricultural Sciences, North-West University, Mafikeng, South Africa; University of Southern California, Los Angeles, California, USA

**Keywords:** endophytes, plant-microbe interactions

## Abstract

Here, we report the draft genome sequence of *Acinetobacter sp*. AYS6, an endophyte isolated from the roots of maize plant in Mafikeng, South Africa. The genome was 7,072,605 bp and exhibited a GC content of 45.6% and 3,654 genes with 3,539 coding sequences, 64 rRNA, 60 tRNAs, and 2 CRISPR.

## INTRODUCTION

*Acinetobacter* spp. are ubiquitous gram-negative, oxidase-negative, non-fermenting coccobacillus belonging to the class of Gammaproteobacteria (1). They live in a variety of ecological niches, including those occupied by humans, animals, and the environment (2). Some researchers have referred to these bacteria as microbial weeds because of their propensity to prevail in so many ecological niches (3). *Acinetobacter sp*. has been widely reported to possess numerous plant-growth-promoting characteristics and be used as a biofertilizer for the improvement of crop productivity and yield (4–7). Several studies have reported the endophytic occurrence of *Acinetobacter sp*. in crops such as tobacco plant roots and rice paddy (8, 9).

*Acinetobacter sp*. AYS6, a non-rhizobial endophyte, was isolated from the roots of *Zea mays* (maize) from Mafikeng, South Africa (25° 47′ 25.24056′′ S 25° 37′30 8.17464′′ E). Healthy maize plants were selected, and their roots were detached and surface sterilized (10–12). The uncrushed sterilized roots were placed on tryptone soya agar (TSA) plates as a control to check the efficacy of surface sterilization. One gram (1 g) of plant material was weighed and macerated. The macerated samples were plated on TSA and incubation at 28°C ± 2°C for 3 days. The single colony was selected and further confirmed through morphological and biochemical tests. To further establish its taxonomy and function, we carried out the whole-genome sequencing of the isolate.

The genomic DNA from the pure culture on solid agar was extracted using Quick-DNA extraction kit (Zymo Research, USA). The genome sequence of strain AYS6 was done at the Novogene Company Limited, Singapore. A paired-end (PE) sequencing library was prepared from the DNA sample using the Illumina Nextera DNA Flex library preparation kit. The PE Illumina library was loaded into the NovaSeq 6000 (2 × 150 bp) instrument for cluster generation and sequencing. Whole-genome sequencing was visualized using KBase (13), which yielded 12,141,328 reads representing; 260 genome coverage. The read quality was examined using Fast QC version 43 1.0.4 (14). Trimmomatic version 1.2.14 was used to filter out the adapter regions and low-quality reads from the data (15). After the filtering and trimming steps, a total of 13,280,536 reads were obtained. The genome assembly was created using SPAdes version v. 3.15.3 (16). Default parameters were used for the software unless otherwise specified. The total assembly size of 7,072,605 bp with 3,420 contigs, 45.6% GC, an *N_50_* value of 157,331 bp, and an *L_50_* value of 8 was obtained. This file was then used as input for GTDB-Tk - v1.7.0 (17) with the final taxonomic output as Acinetobacter sp000313935.

The prediction of gene functions was carried out employing the NCBI Prokaryotic Genome Annotation Pipeline (18). The result revealed a total gene of 3,654 and 3,539 coding DNA sequences, 51 pseudogenes, and 51 noncoding sequences, 64 rRNAs, 60 tRNAs, noncoding RNAs, and 2 CRISPR.

## Data Availability

The whole genome sequence of *Acinetobacter* sp. strain AYS6 has been deposited at DDBJ/ENA/GenBank under the accession number JAOTNV000000000. The version described in this paper is version JAOTNV000000000. The raw reads are available under the BioProject accession number PRJNA882670 and the BioSample number SAMN30948397. The sequence data obtained in this work have been deposited in the NCBI Sequence Read Archive under the accession number SRR21782200.

## References

[1] Adewoyin MA, Okoh AI. 2018. The natural environment as a reservoir of pathogenic and non-pathogenic Acinetobacter species. Rev Environ Health 33:265–272. doi:10.1515/reveh-2017-0034 29982240

[2] Al Atrouni A, Joly-Guillou M-L, Hamze M, Kempf M. 2016. Reservoirs of non-baumannii Acinetobacter species. Front Microbiol 7:49. doi:10.3389/fmicb.2016.00049 26870013 PMC4740782

[3] Cray JA, Bell AN, Bhaganna P, Mswaka AY, Timson DJ, Hallsworth JE. 2013. The biology of habitat dominance; can microbes behave as weeds? Microb Biotechnol 6:453–492. doi:10.1111/1751-7915.12027 23336673 PMC3918151

[4] Suzuki W, Sugawara M, Miwa K, Morikawa M. 2014. Plant growth-promoting bacterium Acinetobacter calcoaceticus P23 increases the chlorophyll content of the monocot Lemna minor (duckweed) and the dicot Lactuca sativa (lettuce). J Biosci Bioeng 118:41–44. doi:10.1016/j.jbiosc.2013.12.007 24468072

[5] Rokhbakhsh-Zamin F, Sachdev D, Kazemi-Pour N, Engineer A, Pardesi KR, Zinjarde S, Dhakephalkar PK, Chopade BA. 2011. Characterization of plant-growth-promoting traits of Acinetobacter species isolated from rhizosphere of Pennisetum glaucum. J Microbiol Biotechnol 21:556–566. doi:10.4014/jmb.1012.12006 21715961

[6] Fadiji AE, Babalola OO. 2020. Exploring the potentialities of beneficial endophytes for improved plant growth. Saudi J Biol Sci 27:3622–3633. doi:10.1016/j.sjbs.2020.08.002 33304173 PMC7714962

[7] Fadiji AE, Babalola OO. 2020. Elucidating mechanisms of endophytes used in plant protection and other bioactivities with multifunctional prospects. Front Bioeng Biotechnol 8:467. doi:10.3389/fbioe.2020.00467 32500068 PMC7242734

[8] Lee J-S, Lee K-C, Kim K-K, Hwang I-C, Jang C, Kim N-G, Yeo W-H, Kim B-S, Yu Y-M, Ahn J-S. 2009. Acinetobacter antiviralis sp. nov., from tobacco plant roots. J Microbiol Biotechnol 19:250–256. doi:10.4014/jmb.0901.083 19349749

[9] Kang Y-S, Jung J, Jeon CO, Park W. 2011. Acinetobacter oleivorans sp. nov. is capable of adhering to and growing on diesel-oil. J Microbiol 49:29–34. doi:10.1007/s12275-011-0315-y 21369976

[10] Vincent JM. 1970. A manual for the practical study of the root-nodule bacteria, p 440. Wiley-Blackwell, Hoboken, NJ.

[11] Fadiji AE, Ayangbenro AS, Babalola OO. 2020. Metagenomic profiling of the community structure, diversity, and nutrient pathways of bacterial endophytes in maize plant. Antonie Van Leeuwenhoek 113:1559–1571. doi:10.1007/s10482-020-01463-w 32803452

[12] Babalola OO, Fadiji AE, Ayangbenro AS. 2020. Shotgun metagenomic data of root endophytic microbiome of maize (Zea mays L). Data Brief 31:105893. doi:10.1016/j.dib.2020.105893 32637499 PMC7327808

[13] Arkin AP, Cottingham RW, Henry CS, Harris NL, Stevens RL, Maslov S, Dehal P, Ware D, Perez F, Canon S, Sneddon MW, Henderson ML, Riehl WJ, Murphy-Olson D, Chan SY, Kamimura RT, Kumari S, Drake MM, Brettin TS, Glass EM, Chivian D, Gunter D, Weston DJ, Allen BH, Baumohl J, Best AA, Bowen B, Brenner SE, Bun CC, Chandonia J-M, Chia J-M, Colasanti R, Conrad N, Davis JJ, Davison BH, DeJongh M, Devoid S, Dietrich E, Dubchak I, Edirisinghe JN, Fang G, Faria JP, Frybarger PM, Gerlach W, Gerstein M, Greiner A, Gurtowski J, Haun HL, He F, Jain R, Joachimiak MP, Keegan KP, Kondo S, Kumar V, Land ML, Meyer F, Mills M, Novichkov PS, Oh T, Olsen GJ, Olson R, Parrello B, Pasternak S, Pearson E, Poon SS, Price GA, Ramakrishnan S, Ranjan P, Ronald PC, Schatz MC, Seaver SMD, Shukla M, Sutormin RA, Syed MH, Thomason J, Tintle NL, Wang D, Xia F, Yoo H, Yoo S, Yu D. 2018. Kbase: the United States department of energy systems biology knowledgebase. Nat Biotechnol 36:566–569. doi:10.1038/nbt.4163 29979655 PMC6870991

[14] Andrews S. 2010. FastQC: a quality control tool for high throughput sequence data. Babraham Bioinformatics, Babraham Institute, Cambridge, United Kingdom.

[15] Bolger AM, Lohse M, Usadel B. 2014. Trimmomatic: aflexible trimmer for Illumina sequence data. Bioinformatics 30:2114–2120. doi:10.1093/bioinformatics/btu170 24695404 PMC4103590

[16] Nurk S, Bankevich A, Antipov D, Gurevich AA, Korobeynikov A, Lapidus A, Prjibelski AD, Pyshkin A, Sirotkin A, Sirotkin Y, Stepanauskas R, Clingenpeel SR, Woyke T, McLean JS, Lasken R, Tesler G, Alekseyev MA, Pevzner PA. 2013. Assembling single-cell genomes and mini-metagenomes from chimeric MDA products. J Comput Biol 20:714–737. doi:10.1089/cmb.2013.0084 24093227 PMC3791033

[17] Chaumeil P-A, Mussig AJ, Hugenholtz P, Parks DH, Hancock J. 2020. GTDB-Tk: a toolkit to classify genomes with the genome taxonomy database. Bioinformatics 36:1925–1927. doi:10.1093/bioinformatics/btz848 PMC7703759 31730192

[18] Haft DH, DiCuccio M, Badretdin A, Brover V, Chetvernin V, O’Neill K, Li W, Chitsaz F, Derbyshire MK, Gonzales NR, Gwadz M, Lu F, Marchler GH, Song JS, Thanki N, Yamashita RA, Zheng C, Thibaud-Nissen F, Geer LY, Marchler-Bauer A, Pruitt KD. 2018. RefSeq: an update on prokaryotic genome annotation and curation. Nucleic Acids Res. 46:D851–D860. doi:10.1093/nar/gkx1068 29112715 PMC5753331

